# Supplementary material for: Patterns of Intron Gain and Loss in Fungi
Source: PLoS Biol. 2004 Nov 30;2(12):e422. doi: 10.1371/journal.pbio.0020422 (PMC532390; doi:10.1371/journal.pbio.0020422)
Supplement: Table S1 — Also available at http://genes.mit.edu/NielsenEtAl/. (4.3 MB ZIP). [file pbio.0020422.st001.zip › NielsenEtAl/html/747.html]

AN6564.1.NCU03827.1.MG06935.1.FG07402.1


```
 CLUSTAL W (1.82) Multiple Sequence Alignments - Introns Inserted


Sequence 1: MG06935.1	307 aa
Sequence 2: FG07402.1	353 aa
Sequence 3: NCU03827.1	314 aa
Sequence 4: AN6564.1	308 aa
Alignment Length: 356 aa
Number Identitical Residues: 101 aa
Alignment Score (without introns) 5372


MG06935.1 	MTTPRAGLA-LLRGCTR--TRRIQWQSLEKGLQALSITPARATIRASSTQAEPMN-----
NCU03827.1	MASLRHSITSALRSSRQGCSKSAQWQSLDQQFGALRISSRSLSTHAHDHNPNVDG--QFI
FG07402.1 	MSTFTQSLR-QLRCQGK--AFRAPWPQPAWQRASFPLAIRTIATETSTSIVEPKPDALAL
AN6564.1  	MHSARSATY--LRAALV--QRSASPLYLAAGAVLPTHTYRHQVTRGYATEVTEAH-----
          	* :   .    **                        :      .               

MG06935.1 	PADAATKG---IAMITHARPLPVSPSYFTLSARFNDDFIKIQNLYRRFQHVPHAPPDKVP
NCU03827.1	AAPALSIDN--FKLHPYARAVPVSPSYFTRTPRFYDNYLSLEKLMEEYEDLPVIPATAVE
FG07402.1 	STDANSKFTMPFQGVDHARAVPLSASYFSREPQFNEMYLRIARLLTKYHALPIVPSSNAP
AN6564.1  	-PAAPEIDFNKVLERRSARVVPASPSYFTGSPRFFDHLLRLENILARYAALPTVAPNEAP
          	 . *       .     ** :* *.***:  .:* :  : : .:  .:  :*  ..  . 

MG06935.1 	RVAWRKFQDYRAFCGEAVRASHYSECIAMAKHLHKIHPDLKSEEVVQALHPFKRDINAHG
NCU03827.1	RVAWKTLEDIRKELGEQVKASEFARCLALVKRLNSIHPDLKPQEIKDVLDSFRRNVQPFT
FG07402.1 	PTAWVKLQEMRSKLGEPIKASHYAKVIRVAKRLNQIEPSLFPEEVRAALEEFKRDINPFM
AN6564.1  	RMAWLKLPAFREFVGERVPTKKYKGLIKVLQRLNRINPDILPDEVRRALKDFLRPGNPYG
          	  ** .:   *   ** : :..:   : : ::*: *.*.: .:*:  .*. * *  :.. 

MG06935.1 	NAVRTIPIDKFGRALGTGRRKTSVARAWVVEGSGEIIINGKSLAEAFGRVHDRESAIWAL
NCU03827.1	NVAKPIPIDQFGRACGVGKRKASSARAFVVEGTGEVLVNGKTLAEYFGRVHDRESAVWAL
FG07402.1 	NVPHEVIIDKYGRAVGVGKRKESTARAWVVEGTGEVLINGKTLSQAFGRVHDRESAVWAL
AN6564.1  	TQSITTTVDELGRARGKGKRKESSAVVSLVEGDGEVRVNGKTLVEAFPRVHDRESATWAL
          	.      :*: *** * *:** * * . :*** **: :***:* : * ******** ***

MG06935.1 	RASQRLDKYNVWALVEGGGPTGQAEAMTLAVAKALMAHEPALKPALRRA1GCVTRDPRMV
NCU03827.1	RATNRIDKYNVWVKVEGGGTTGQAEAITLAIAKALLAHEPALKPALRRA1GCVTRDPRKV
FG07402.1 	QATERLDKYNVWALVEGGGTTGQAEALTLAVAKALIVHEPALKTALRKA1GCITRDPRTV
AN6564.1  	RCSNRLDKYNVWATVKGGGTTGQAEALALALGRALMIHEPALKPILRRA1GVITVDARRV
          	:.::*:******. *:***.******::**:.:**: ******. **:* * :* *.* *

MG06935.1 	ERKKPGHVKAR~-------------~-------------------------KMPAWVKR
NCU03827.1	ERKKHGHVKAR~-------------~-------------------------KMPTWVKR
FG07402.1 	ERKKHGHVKAL1CNGGYDDDDEKDT1VGRYTAATDSEPDGQITSNSLFGLSEIPAAWDP
AN6564.1  	ERKKPGHVKAR~-------------~-------------------------KSPTWVKR
          	**** *****                                         : *:  .
```
